# Recent advances of chimeric antigen receptor T‐cell therapy for acute myeloid leukemia

**DOI:** 10.3389/fimmu.2025.1572407

**Published:** 2025-05-02

**Authors:** Yang Liu, Wanting Wang, Chaofan Wang, Jun Deng, Yu Hu, Heng Mei, Shanshan Luo

**Affiliations:** ^1^ Institute of Hematology, Union Hospital, Tongji Medical College, Huazhong University of Science and Technology, Wuhan, China; ^2^ Key Laboratory of Molecular Biological Targeted Therapies of the Ministry of Education, Wuhan, China

**Keywords:** acute myeloid leukemia, chimeric antigen receptor T cell, immunotherapy, adoptive cell therapy, immunosuppressive microenvironment

## Abstract

Acute myeloid leukemia (AML) is a heterogeneously primary hematopoietic neoplasm characterized by uncontrolled proliferation of immature myeloid cells, which is characterized with poor outcomes. Despite tremendous advances in the treatment paradigm of AML in the past several decades, the cure and prognosis remain unfavorable. More effective treatments are therefore needed to improve the clinical outcomes. Among newly emerging immunotherapies, chimeric antigen receptor (CAR)-T cell immunotherapy is an exceedingly promising approach that has remarkably improved the overall survival for patients with AML. However, current CAR-T cell therapy for AML faces numerous significant challenges such as the identification of truly AML-specific surface antigens, the on-target/off-tumor toxicity, and the immunosuppressive microenvironment of AML. In order to conquer these limitations, novel strategies to advance CAR-T therapy are urgently needed. In this comprehensive review, we summarize the current status of immunotherapy, especially CAR-T cell therapy, highlight the outcomes of current trials and the limitations of CAR-T immunotherapy, hopefully to provide novel insights into the future directions of CAR-T cells in AML.

## Introduction

In recent years, the occurrence of AML has been increased annually. It is reported that the incidence of AML in the US in 2023 is 20380 cases with approximately 11310 deaths (1, 2). Currently, the combination of cytarabine (ara-C) and anthracycline remains the standard induction chemotherapy for AML, which is commonly known as the “3 + 7” regimen, resulting in long-term cures of approximate 35% of the younger AML patients (3). Small molecular drugs that target specific molecules are gaining attention for their potential in treating AML. Among these, some are already used in clinic such as ivosidenib (IDH1 inhibitor) (4), enasidenib (IDH2 inhibitor) (5), gilteritinib (FLT3 inhibitor) (6), and venetoclax (BCL-2 inhibitor) (7). Despite the advancements of current available therapies, the majority of patients still have terrible prognosis due to the disease progression or recurrence, as a result of treatment resistance or adverse side effects (8, 9). Therefore, it is essential to identify and research potential novel therapeutic approaches for AML.

T-cell-based immunotherapy is an effective strategy, including the genetic modification and redirection of these cells to eradicate AML blasts (10). For example, CAR-T cell therapy is a relatively novel strategy, in which autologous/allogeneic T cells are collected and reprogrammed to express CARs that recognize tumor surface antigens specifically. The reprogrammed T cells can specifically identify tumor-associated targets and destroy these cells without the assistance of the major histocompatibility complex (11, 12). The treatment of hematological malignancies is the primary area for CAR-T cells, which has shown an impressive overall and complete response rate. This is because the adequate tumor antigen is easier to find and target in hematological malignancies compared with solid cancers (13, 14). However, due to the significant genetic and phenotypic heterogeneity, finding a true AML-specific antigen is challenging, which limits the successful application of CAR-T cell therapy in AML treatment (15). In addition, the expression of AML antigens on normal healthy tissues often causes variable degrees of toxicity due to the mistarget of the healthy tissues. The on-target/off-tumor toxicities are usually unavoidable for CAR-T therapy, such as the possibility of fatal myeloablation when targeting myeloid precursor cells (16). Here, we outline the progress achieved in the multiple categories of immunotherapeutic approaches for the management of AML, further discuss the particular mechanisms of CAR-T therapy, summarize the recent advances of CAR-T immunotherapy in AML, as well as the current limitations, hopefully providing some novel insights for the future research direction.

## Overview of the current available immunotherapies for AML

In the management of AML, current available immunotherapy involves targeted antibodies, adoptive cell therapy (ACT), immune checkpoint inhibitors (ICIs), Hematopoietic stem cell transplantation (HSCT), and tumor vaccines. Among them, the allogeneic HSCT is still one of the most classic and effective immunotherapeutic approaches for hematological malignancies (17, 18). As the understanding of the genetic and phenotypic diversity of AML rapidly advances, immunological therapeutic targets have been revealed increasingly. Over the past 10–15 years, several small molecule targeting drugs have been successfully used for AML, either alone or in a combined form with other standard therapies (19, 20). For example, midostaurin which inhibits multiple tyrosine kinase receptors, is approved for FLT3-mutated AML alone or combined with chemotherapy (21). Cancer vaccines are a positive strategy to eliminate AML and prevent tumor recurrence, which stimulates a persistent immune response. Recently, researchers have developed the ECNV-αGC vaccination, demonstrating its efficacy in reducing the burden of AML. However, there is still a long way to go before cancer vaccines can be translated from the bench to the bedside (22). Despite the numerous ongoing trials, these immunotherapies for AML still have many limitations to overcome.

ACT has emerged as a widely applied immunotherapeutic strategy for patients with AML, including DC cell, TCR-T cell, cytokine-induced killer (CIK) cell and CAR-NK cell. Among them, CAR-T cells have become a promising candidate (23). The remarkable successful outcomes of CAR-T in other hematopoietic malignancies, such as acute lymphoblastic leukemia (ALL), have prompted their attempted application in AML (24). In a phase I clinical trial involving 10 cases, CLL-1 CAR-T cells demonstrated remarkable therapeutic potential that 7 of 10 R/R AML patients achieved CR/CRi (25). Although the application of CAR-T therapy in AML treatment remains in the early stages, trials to date have achieved encouraging initial outcomes just like in other hematological malignancies.

## Principles of CAR-T cell therapies

**Figure 1:**
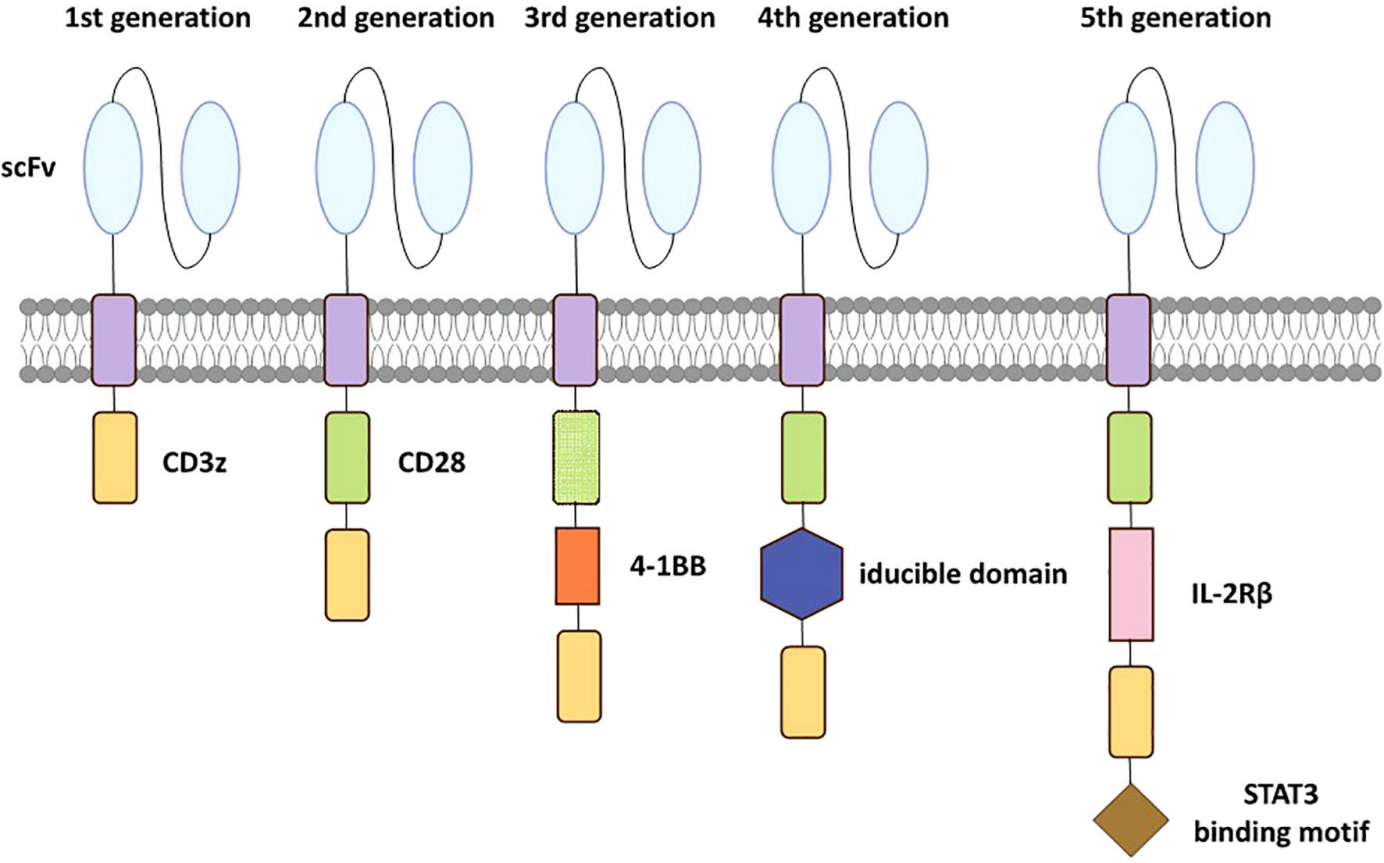
Evolution of the 5 generations CARs. The first-generation has only a CD3z signaling domain. The second-generation is characterized by an additional costimulatory domain based on the first-generation. The third-generation incorporates two costimulatory domains similarly. The fourth-generation has an additional inducible domain to induce the production of tumor-killing cytokines. The fifth-generation incorporates an intracellular IL-2Rβ domain with a STAT3 binding motif to activate the JAK-STAT path.

Although the detail structures of each CAR construct are slightly different, the most commonly used CARs include an antigen binding domain, an activation domain from CD3z, an extracellular hinge domain and an intracellular costimulatory domain (**Figure 1**). When CAR-T cells recognize and bind to tumor-specific antigens, the intracellular structural domain initiates activation procedures via phosphorylation and subsequent signaling. Consequently, CAR-T cell specifically targets tumor-associated antigens with HLA independence (11). The primary cytotoxicity hinges on the cytokines secreted by CAR-T cells including granzymes and perforins (26). Furthermore, CAR-T cell triggers the apoptotic signaling cascade through the engagement of surface molecules, ultimately leading to the programmed death of cancer cells (27). The efficiency of the first generation was disappointing due to the absence of co-stimulatory signaling function. Therefore, the second‐generation CAR construct has one additional co‐stimulatory domain that differs from the first, respectively. Such bi-co-stimulatory domains enhance the activation and proliferation of CAR T cells. Furthermore, the structure of the third generation is similar to the second (28), while the fourth generation CAR-T has an additional inducible domain. When the domain is activated, CAR-T cells generate numerous cytokines displaying anti-tumor activity in local tumor tissue, leading to the improvement of durability and other comprehensive anti-tumor responses (29). The fifth generation incorporates an additional intracellular IL-2 receptor domain into the design of the second generation. This modification induces the production of memory T cells by the antigen-driven activation of the JAK/STAT signaling pathway, improving the therapeutic efficacy of CAR-T therapy. Genome editing technology is also used in the fifth generation, aiming to mitigate the risk of cytokine release syndrome (CRS) by inhibiting dominant negative receptors, including PD-1 and TGF-β (30).

Current CAR-T production methods mainly involve ex vivo strategy which needed isolation, genetic modification, and subsequent expansion of T cells outside the body, as well as *in vivo* strategy that engineered CAR-T cells directly within the body using delivery vehicles (31). Despite the pre-clinical results showing that *in vivo* production is less complicated than ex vivo production, *in vivo* CAR-T production is still under research, with no product receiving approval from the FDA compared with the mature ex vivo production strategy (32, 33).

## Current CAR-T cell constructs for acute myeloid leukemia

CAR-T cell therapy is still in the early stages in AML compared with other hematopoietic malignancies. Currently, no CAR-T product is approved for clinical use in AML, however numerous AML-directed CAR-T cells are developed in preclinical and clinical trials. To designing an effective CAR, choosing an appropriate target is the most critical step. However, most of the targets identified in AML cells have not been ideal until now. The predominant targets of current clinical trials of AML are CD33 and CD123, however, these identified targets may also be expressed on healthy HSCs or may not be consistently presented in all AML cells. Currently, in a phase clinical I study, CLL-1 CAR-T cells in the R/R AML patients show a promising outcome with the CR/CRi rate 70% (n = 7/10), but off-target toxicity is quite concerning (25).

To overcome the limitations of CD33 and CD123 targets, researchers are exploiting other novel targets, such as NKG2DL, CLL-1, CD70, CLEAC12A and CD138, as well as dual antigen‐directed CARs. In the following sections, we will summarize the targets under current research (**Table 1**), and discuss the corresponding advances and the challenges of CAR-T cell therapy.

**Table 1:** Selected landmark clinical trials of chimeric antigen receptor T‐cell therapy in acute myeloid leukemia.

Targets	CAR	ScFV origin	Cosignaling domain	Outcomes	Reference
CD33 and CD123	Dual CAR	Human	CD28 and 4-1BB	Reduced tumor burden (BM: 0.06% vs. 65.7% in controls, P < 0.05) prolonged survival *in vivo* (mice) low toxicity to endothelial cells and HSPCs.	(34)
CD33	DARIC33	Llama	4-1BB	Phase I clinical trial initiated (PLAT-08, NCT05105152)	(35)
CD123	CD123 CAR	Human	CD28 and OX40	Enhanced anti-AML activity *in vivo* with AZA pre-treatment	(36)
CD123	UCART123	Murine	4-1BB	2.5×10^6 UCART123 cells significantly extended overall survival in PDX-AML2 and PDX-AML37 models	(37)
CD123 and NKG2DLs	123NL CAR	Human	4-1BB	Mice with chloroform-labeled tumor cells had a 90% higher survival rate compared to controls	(38)
CD7	CD7 CAR	Human	CD28	In the xenograft model, there was no tumor growth at 125 days (median survival of control group: 54 days)	(39)
CD7	CD7 CAR	Murine	4-1BB	Tumor load was no longer detectable at day 22 after injection in xenograft mice	(40)
CD7	CD7 CAR	Murine	CD28 and 4-1BB	Patient with relapsed/refractory AML achieved MLFS (bone marrow blasts: 20% to 0%)	(41)
CD7	CD7 CAR	Human	4-1BB	At 28 days post-infusion, 81.8% (9/11) had objective responses, including a complete response rate of 63.6% (7/11)	(42)
CD117	CD117 CAR	Human	4-1BB	CD117+ tumor cells are killed in a humanized mouse model	(43)
CD70	CD70 CAR	Human	CD28 and 4-1BB	In xenograft models, CD27z-CAR T cells induced complete leukemia remission in all xenograft mice by day 21	(44)
CD70	CD70 CAR	Human	4-1BB	Prolonged survival in mouse models (median survival extended by about 50%)	(45)
CD93	NOT-gated CD93 CAR	Human	CD28 and 4-1BB	Complete remission in 80% of mice with CD93–28z and 70% with CD93–BBz in PDX model (N=17 for CD93–28z, N=20 for CD93–BBz)	(46)
CD38	CD38 CAR	Human	4-1BB	ATRA treatment increased CD38 expression to 99.94% and specific cytotoxicity to 98.92%.	(47)
FLT3	FLT3 CAR	Human	CD28	NSG mice carrying MOLM-13 AML cells had a higher overall survival rate compared to controls (p<0.05)	(48)
FLT3	FLT3 CAR	Human	4-1BB	Complete and durable responses in mice with low disease burden	(49)
CD44v6	CD44v6 CAR	Human	4-1BB	Significantly inhibited tumor progression in FLT3 or DNMT3A mutant AML cells in a mouse model	(50)
CLL1	CLL1 CAR	Murine	CD28 and 4-1BB	Significantly reduced leukemia burden and improved survival in xenografted mice (p < 0.001)	(51)
CLL1	CLL1 CAR	Human	CD28 and CD27	3 achieved CR with MRD negativity. One patient survived for 5 months.	(52)
CLL1	CLL1 CAR	Murine	CD28 and OX40	Molecular CR achieved in 2 post-transplant relapse patients, with sustained remission of 8 and 3 months at last follow-up	(53)
CLEC12A and ADGRE2	ADCLEC.syn1	Human	4-1BB	2 out of 2 AML PDX models showed complete remission	(54)
FRβ	HA FRβ CAR	Human	CD28	Exhibited enhanced anti-leukemic activity *in vitro* and *in vivo* compared to LA FRβ CAR T cells	(55)
CD123 and FRβ	Bispecific TanCAR	Human	CD28	Produced higher levels of IFN-γ and IL-2 than monospecific CAR-T cells.	(56)
GRP78	GRP78 CAR	Human	CD28	Prolong survival (p < 0.0001) but appeared to relapse	(57)
NKG2DL	NKG2DL CAR	Human	Dap10	Median OS was 4.7 months (range, 1.2–24.9+ months) with 2 patients alive at 16.8 and 24.9 months.	(58)
FLT3 and NKG2DL	FLT3scFv/NKG2D CAR	Human	4-1BB	Gilitinib pretreatment prolonged median survival from 24 days with monotherapy to 35 days with combination therapy	(59)
LILRB3	LILRB3 CAR	Human	4-1BB	All treated mice achieving lasting remission and survival exceeding 100 days (n=7)	(60)
PRAME	PRAME mTCR CAR	Human	4-1BB	In the THP-1 model, median survival was extended to 110 days	(61)

PDX, patient-derived xenograft; HA, High Affinity; FRβ, Folate Receptor β; TanCAR, Tandem Chimeric Antigen Receptor.

### CD33

CD33 is an immunoglobulin-like lectin which expressed on cells of the monocytic and myeloid lineages, and is present in 87.8% of AML cases (62). However, the expression of CD33 in healthy HSCs was also detected, which could lead to off-target toxicity (63). Taking this into account, researchers performed an *in vivo* experiments through xenograft mouse model to selectively delete CD33 from normal HSCs to avoid undesired toxicity. Consequently, CAR-T cells targeting CD33 could efficiently destroy AML cells without myelotoxicity (64). Furthermore, a more commonly used strategy is to establish a balanced dual-CAR. Researchers established a balanced dual-CAR based on a low-affinity interleukin-3-zetakine (IL-3z) and a high specificity of CD33 to target AML cells. This CAR is designed without activating signaling domains in order to minimize off-target toxicity and maintain complete damaging capacity against AML cells (34). Meanwhile, the design of CAR that incorporates pharmacologic control is also considered. A clinical trial with rapamycin-regulated CD33 CAR-T cells has shown a controlled function in AML, demonstrating a promising prospect (35).

### CD123

CD123, a cell membrane protein, is notably overexpressed on AML cells, but nearly absent on normal HSCs (65),unlike CD33, but CD123 expression on blood vessels leads to off-target toxicity (66). Researchers designed the CD123 CAR-T cells and analyzed the therapeutic effects on AML in a xenograft model. The data showed that these CAR-T cells exhibited anti-AML activity, importantly without toxicity to the hematopoietic system or other tissues (36). Furthermore, researchers found that 5′-Azacitidine (AZA) treatment could increase the density of CD123 on AML cells, therefore enhancing activity and abundance of CTLA-4^neg^ CAR-T cells, revealing the potential benefits of combining CAR-T therapy with pharmaceuticals (36). A study evaluated the therapeutic effects of allogeneic gene-edited CAR-T cells (UCART123) targeting CD123 both *in vitro* and *in vivo*, displaying high efficacy to eliminate AML cell. In addition, the safety features are impressive by using genome editing technology to avoid graft versus host disease and expressing special antigens to eliminate CAR-T cells timely (37). Similarly, researchers also developed the bispecific CAR-T cell for both CD123 and NKG2DL, that not only eliminates AML cells but also targets immunosuppressive cells. Consequently, this dual-CAR strategy perfectly avoids antigen escape and counteracts the suppressive impacts of tumor microenvironment (TME) (38).

### CD7

As a surface marker, CD7 is commonly expressed on T lymphocytes and natural killer cells (67). It is also expressed in about 30% of AML cases, but is absent on healthy myeloid cells, leading to high specificity and limited toxicity (68). Considering its expression on T cells has been proving fratricidal, researchers used genome editing technology to produce the CD7^KO^ T cells that effectively eliminate CD7 AML cells but spare healthy myeloid cells (39). Later, the specific CD7 CAR-T cells which have a low expression of CD7 was further designed by transducing an anti-CD7 CAR into T blasts followed by the natural selection. These cells inhibited leukemia cell proliferation in a xenograft mouse model and efficiently killed CD7 AML cells of R/R AML patients *in vitro*, showing a novel effective strategy without expensive gene ablation (40, 69). A clinical trial reported a R/R AML patient who was treated with CD7 CAR-T cell therapy, displayed reduced tumor burden with controlled CAR-related toxicity (41). Another study also tested the therapeutic effects of CD7 CAR-T cells (RD13-01) in a R/R AML patient which achieved an MRD++− CR after CD7 CAR-T treatment (42).

### CD117

CD117, also recognized as KIT, is categorized as a type III receptor tyrosine kinase, which is predominantly expressed on the majority of myeloid blasts and is crucial in the AML development. Nevertheless, the expression of CD117 is significantly elevated on healthy HSCs and most primary AML cells (70). Therefore, the CD117 CAR-T therapy which targets both healthy and cancerous cells at the same time to avoid additional myeloablative conditioning before HSCTs, holds promise as a transitional treatment of HSCTs. A study had successfully generated and examined CD117 CAR-T cells in a xenograft mouse model, showing an efficient clinical prospect (43).

### CD70

CD70 is a tumor necrosis factor (TNF) superfamily member and is expressed on most leukemic blasts, but unlike CD33 and CD123, it is absent from normal HSCs (71). In a conducted research, researchers produced a series of CD70 CAR-T cells and evaluated their activity against AML both *in vivo* and *in vitro*. The findings indicated that the CAR utilizing the CD70 receptor CD27 had an enhanced anti-AML activity when compared to the conventional scFv-based CAR-T cells (44). In another study, another CD70-targeted CAR was designed which has a panel of hinge-modified regions. Functional analysis showed an enhanced ability to target tumor surface antigens (72). Researchers also evaluated CD70 targeted CAR-T in xenograft mouse model, exhibiting that such CAR-T displayed significant anti-AML activity and durability in an xenograft mouse model (45).

### CD93

CD93 is a transmembrane glycoprotein, which is predominantly expressed in AML blasts and LSCs, but is absent in healthy HSCs (73).Importantly, CD93 expression is relatively stable and highly expressed in a significant proportion of relapsed AML patients. Therefore, CD93 is a perfect target for CAR-T cell therapy. Researchers have developed CD93 CAR-T cells utilizing a humanized CD93-specific binder which effectively targets and eliminates AML cells without side toxicity to HSCs. Furthermore, they introduced the NOT-gated CD93 CAR-T cells, aiming to overcome the undesired endothelial-targeting toxicity (46).

### CD38

CD38 is expressed on most leukemic blasts and has successfully been harnessed in the treatment for various hematological malignancies, including multiple myeloma and ALL (62, 74). Considering the relatively low expression of CD38 in AML, a study was conducted to test its anti-AML effects when enhancing CD38 density on AML cells by combining all-trans retinoic acid (ATRA) with CD38 CAR-T cells. The research data demonstrated that ATRA significantly enhanced the anti-AML activity of CD38 CAR-T cells through elevating the CD38 surface expression levels (47). Recently, researchers developed and evaluated a novel CD38-targeting T-cell engager, which showed an enlightening outcome. The outcome demonstrated that this CD38-targeting T-cell engager could stimulate T cells to release IFN-γ and transform surrounding CD38^neg^ cells into CD38^pos^ cells when interacting with CD38^pos^ AML cells, thereby efficiently eliminating AML. This strategy showed good application prospect, which may be used in the construction of CAR in the future (75).

### FLT3

FMS-like tyrosine kinase 3 (FLT3) is typically expressed both on healthy HSCs and on AML blasts, with high specificity for FLT3 with internal tandem duplication (FLT3-ITD) (76). A study examined FLT3-specific CAR-T cells, revealing that these CAR-T cells could recognize and disrupt healthy HSCs *in vitro* and *in vivo* (48). Therefore, researchers further improved and developed an allogeneic CAR-T cell with the elimination of endogenous TCR, achieving a lower risk of alloreactivity and a more timely treatment with off-the-shelf CAR-T cells (49). In addition, considering about 37% AML patients have FLT3 mutations and a high expression of CD44v6, researchers constructed CD44v6 CAR-T cells to treat these patients with FLT3 mutations (50).

### CLL1

The human C-type lectin-like molecule-1, identified as CLL-1 or CLEC12A, is primarily expressed on most AML blasts. Importantly, CLL-1 is expressed within LSCs contrasting with its absence in HSCs, much like CD123 (77). The CLL-1 CAR-T cells have been proven to specifically damage AML cells *in vitro* without toxicity to HSCs (51). A study also identified CD33/CLL1 as the preferred combinatorial targets for pediatric AML, which further expanded the potential clinical application of CAR-T (52, 78). 2 patients with R/R AML displayed successful outcomes when treated with PD-1 silenced CLL-1 CAR-T therapy, after the failure of HSCT and CD38 CAR-T therapy (53). In a clinical trial, CLL-1 CAR-T therapy showed positive efficacy and tolerable safety in R/R AML patients (25). Furthermore, researchers developed a special CAR that combined the CLL1 as a costimulatory receptor with the ADGRE2-CAR to specifically target ADGRE2^pos^ and CLL1^pos^ LSCs, while sparing the ADGRE2^low^ and CLL1 ^neg^ healthy HSCs. Collectively, this combined targeting strategy could selectively eliminate AML cells and reduce hematological toxicity (54).

### Folate Receptor β (FRβ)

In 2015, the first production of FRβ CAR-T cells which selectively disrupted AML cells, showed therapeutic potential. Furthermore, the application of ATRA resulted in improved elimination of AML cells with enhanced FRβ expression (79). A subsequent study proved that the high-affinity FRβ CAR-T cells displayed greatly enhanced anti-tumor activity compared with the low-affinity FRβ CAR-T cells (55). Furthermore, researchers generated a bispecific tandem CAR by combining FRβ with CD123 in the retroviral vector, proving to have an enhanced effect for AML (56).

### GRP78

Glucose-regulated protein 78 (GRP78) is typically located within the endoplasmic reticulum (ER). However, when ER stress is elevated, the overexpressed GRP78 is transferred to the surface of tumor cells (80). Researchers designed T cells expressing a peptide-based CAR specifically targeting GRP78, and proved a decrease in fratricide treated with dasatinib during the production. In addition, the GRP78 CAR-T cells could effectively eliminate GRP78^pos^ tumor cells without toxicity against HSCs (57).

### NKG2DL

Natural killer group 2D ligand (NKG2DL) is widely expressed in various malignant neoplasms, but nearly absent in healthy tissues (81). In a phase I clinical trial, a single patient who received the maximum dose of NKG2D CAR-T cell therapy proved to have an improvement of blood cell counts and maintained clinical stability over several months without additional supplementary treatment. Since the endpoints of this clinical trial are assessing the feasibility and safety of a single injection of NKG2D CAR-T cells instead of stable disease, no objective clinical efficacy of CAR-T cells was proved. However, considering the outstanding safety and the unexpected disease stability of several patients during subsequent therapies, NKG2D CAR-T cells have shown potential therapeutic value in AML (58). Since high expression of NKG2DL can be induced by FLT3 inhibitors, researchers constructed dual-target FLT3scFv/NKG2D CAR-T cells, and examined the inhibitory effects *in vitro*, which showed the powerful ability to lyse AML cells and improvement by gilteritinib-pretreatment (59).

### LILRB3

The members of LILR subfamily B (LILRB) are negative factors to regulate myeloid cell activation and are commonly expressed on myeloid and lymphocyte cells (82). In a recent study, CAR-T specifically targeting LILRB3 has exhibited remarkable anti-AML activity both *in vivo* and *in vitro*. Furthermore, LILRB receptors are upregulated in response to inflammatory stimulus and chemotherapy conditions, suggesting that combined CAR-T with specific stimulus can be applied by artificially regulating the tumor microenvironment to improve the LILRB3 CAR-T efficacy (60).

### Intracellular targets: PRAME

PRAME is a melanoma-associated antigen overexpressed in a variety of hematologic malignancies, including AML and CML. Conventional CAR-T cells are unable to target PRAME because it is an intracellular antigen (83). Researchers developed a special CAR-T (PRAME ^mTCR^CAR-T) by using a T-cell receptor mimic antibody that recognizes the complex constituent of HLA-A2 and PRAME ALY peptide, therefore achieving an effective anti-AML capacity upon applications of such CAR-T cells *in vivo* (61).

## Limitations of CAR-T cell therapy in the treatment of AML

Despite the above-mentioned examples of CAR-T cells in AML, many challenges existing limit the clinical efficacy of CAR-T cells in AML. Limitations to effective CAR-T cell therapy include restricted anti-tumor efficacy, severe life-threatening toxicities, relapse and resistance. Furthermore, other unsolved challenges commonly exist. For example, the tumor microenvironment significantly influences the activity of CAR-T cells. The excessive period of waiting for treatment initiation, the requirement for an optimal CAR design, the design of the most effective intracellular costimulatory domains, and the determination of the optimal timing for CAR-T cell infusion are all critical and unsolved for CAR-T therapy in AML.

### Restricted anti-tumor efficacy

Although AML has a modest mutational load in contrast to other malignancies like melanoma or lung cancer, the genetic diversity, epigenetic alterations, and clonal heterogeneity all contribute to the complexity of CAR-T therapy in AML. Among them, the genetic and phenotypic heterogeneity in the AML cells is the foremost challenge that limits the applicability of the universal CAR-T cells (84). Currently, the resistance mechanisms of CAR-T therapy remain largely elusive, only with some hypotheses including tumor heterogeneity, antigen escapes, and the exhaustion of T-cells, along with their diminished persistence. However, it is evident that the primary forms of resistance involve antigen-negative and antigen-positive relapses. These antigen-negative relapses are linked to a range of factors, such as CAR-T cell-induced mutations, alternative splicing, the masking of epitopes and low antigen density (85–87). In a study utilizing a mouse model of leukemia, it has been demonstrated that target antigens can be transferred to T cells via CAR T cell trogocytosis. This process results in a reduction in the density of target antigens on tumor cells, thereby promoting the exhaustion of CAR-T cells. Co-targeting different antigens may prove beneficial in addressing this type of antigen-negative relapses. Antigen-positive relapses are frequently attributed to inadequate persistence of CAR-T cells, which might be caused by several factors, including the immunogenicity of the CAR itself, the inherent quality of the T-cells, the initial phenotype of the T-cells, the co-stimulatory domain present within the CAR constructs, and the impact of the tumor microenvironment (88–90).

Other significant factors contributing to the diminished efficacy of CAR-T therapy are inadequate T cell proliferation and short-term T cell survival, which leads to a weak therapeutic response. It is widely believed that the immunosuppressive microenvironment created by AML contributes much to such restrictions. Among the critical elements for the suppressive tumor microenvironment, regulatory T (Treg) cells play a prominent role in inhibiting immune responses. First, Tregs with an overexpression of PD, OX40 and TIM3 have an increased frequency in the peripheral blood of AML patients (91). The engagement of PD-1 with PD-L1 or PD-L2 initiates a series of intracellular signals that inhibit T-cell activation. Furthermore, the expression of PD-1 on Tregs that migrate to the tumor microenvironment can stimulate these immunosuppressive cells, reinforcing their immunosuppressive functions (92, 93). Recently, the 123NL CAR-T cells likely targeting immunosuppressive cells through CD123 and NKG2DL, demonstrated a promising approach to overcome tumor microenvironment (38). In addition, researchers have proved that TP53 deficiency confers resistance of AML. Therefore, inhibiting the mevalonate pathway in TP53-deficient AML cells or enhancing the Wnt pathway in CAR-T cells *in vitro* restores the efficiency of CAR-T-cell-mediated AML cell lysis in a recent study. This data provided a novel insight into our understanding of CAR-T therapy resistance in terms of metabolic mechanisms (94). Lots of studies have been done to overcome these immune pathways which were hypothesized as the important barriers to T cell activation, but the conclusion remains uncertain.

### Severe life-threatening toxicities

The frequently observed adverse effects for CAR-T therapies are CRS, neurotoxicity, cytopenias and infections. These adverse effects can range from minor symptoms to serious life-threatening situations. For example, ten R/R AML patients treated with CLL-1 CAR-T cells all suffered from CRS, among them, 4 cases of them were low-grade, the remaining 6 were considered high-grade (25). The precise mechanism of CRS remains elusive but is theorized that it may arise from the over-activation of T-cells, which subsequently leads to the emission of a variety of inflammatory cytokines such as TNF-α and interferon-gamma (95). Symptoms associated with CRS triggered by CAR-T cell therapy may include fever, tachycardia, headache, nausea, rash and shortness of breath. Furthermore, some severe cases could even lead to multiple organ failure (96). The standard approach to managing CRS typically involves supportive care alongside the administration of corticosteroids or tocilizumab as treatment options (95, 97).

Despite the enhanced targeting precision that CAR-based therapies present in comparison to conventional chemotherapy and radiotherapy, the on-target/off-tumor toxicity remains common and troublesome. Such type of toxicity is characterized by the devastation of health tissue when targeting tumor antigens due to the common expression of target tumor antigens on healthy cells. On-target/off-tumor toxicity is a common issue in CAR-T cell therapies, but many of its manifestations remain unidentified or are also obscured by other symptoms. For example, some toxicities are directly associated with the targeting effects by CAR-T cells, such as immune effector cell-associated neurotoxicity syndrome, hypogammaglobulinemia, hematologic toxicities; the others may be indirectly linked to the therapy-induced immunosuppression to the patients, such as infections, sepsis (98).

## Potential effective strategies to improve CAR-T treatment, specially for failure and resistance

CAR-T cell therapy has achieved numerous encouraging progress in AML. This therapy has inimitable advantages compared with conventional chemotherapy and radiotherapy, but also has some limitations that need to be addressed. A variety of strategies including the combination of CAR-T cell therapy with other anti-AML approaches and the utilization of advanced CAR engineering techniques, have been put forward to enhance the therapeutic efficacy of CAR-T, mitigate adverse effects and broaden clinical applicability. In the following section, we describe some strategies for overcoming these limitations.

### Optimizing CAR structure

The current clinical outcomes of CAR-T cells are unsatisfactory for AML therapy due to the modest cell responses. To improve it, researchers are extremely enthusiastic about modifying the structure of CAR. Recently, research has reported that a CD33 CAR targeting membrane-proximal epitopes outperforms the CAR targeting membrane-distal epitopes, emphasizing the importance of the antigen epitopes for optimizing CAR design (99). Researchers redesigned a series of hinge domains to reduce the proteolytic cleavage of the CD27 extracellular segment. This modification enhanced the binding stability between the CAR to CD70, thereby enhancing the binding ability and anti-tumor activity *in vivo* (72). Continuous optimization of the CAR structure might overcome the root cause of the undesired efficacy of CAR-T cells in AML. In addition, using different target-specific CARs coherently may also be a promising strategy. Researchers established a novel T-cell platform known as the Fab-based adapter CAR (AdCAR) which uses adapter molecules such as anti-CD33, anti-CD123, and anti-CLL1 to selectively target AML cells. The AdCAR platform overcomes the chronic T-cell exhaustion and antigen heterogeneity in AML, providing a more adaptable strategy against the complexities of AML (100).

Targeting at a single antigen may produce a selective pressure which potentially drives the evolution of cancer cells and results in the immune escape. Therefore, bispecific CAR-T cell is a promising therapeutic approach for AML. The bispecific CAR-T cells targeted both GRP78 and CD123 had been proven to successfully improve anti-AML activity compared to the CAR-T cells targeted GRP78/CD123 (101). A vitro study has demonstrated that CAR’TCR-T cells, engineered to co-express both a CD33-CAR and a transgenic dNPM1-TCR, exhibit enhanced and sustained anti-tumor efficacy. This is especially notable in a case where the target antigen density is extremely low, highlighting the potential value of this dual-expressing cell strategy (102). In addition, discovering novel CAR targets is also meaningful for the therapy efficacy of CAR-T. A study proposed an approach that evaluated many candidates simultaneously and applied some particular principles to guide combinatorial pairings (103). Structural surface-omic and Single-cell transcriptomic might also be helpful for discovering new targets (90, 104). Given the current lack of sufficiently suitable targets, a strategy of targeting multiple antigens may represent the optimal approach to overcome the heterogeneity of AML. Continuous refinement of CAR structures through bioengineering techniques, coupled with the potential of gene editing to enhance CAR-T efficiency, holds promise for overcoming AML heterogeneity.

### Combining other therapies to improve the efficacy of CAR-T

Combining CAR-T cell therapy with other therapy is also necessary to improve the anti-tumor efficacy in AML. Research has confirmed that the pretreatment with rapamycin, which diminishes mTORC1 signaling, can attenuate the activity of CAR-T cells to infiltrate bone marrow by diminishing mTORC1 signaling. This intervention has been shown to intensify the eradication of AML cells within the bone marrow in mouse models of leukemia xenografts and may inspire us to combine other chemotherapeutic agents with CAR-T cells for AML treatment (105). Furthermore, a study reported a CD38-CD3 T-cell engager, named BN-CD38 was developed, which has demonstrated the ability to facilitate T-cell activation and proliferation, as well as contribute to the elimination of AML LSCs within an autologous context, offering a promising strategy for the targeted treatment of AML. Interestingly, IFN-γ–induced upregulation of CD38 may improve the CAR-T therapy through higher antigen density (75). Combining CAR-T therapy with AZA (demethylating agents) appears highly promising for clinical translation (36). Additionally, integrating CAR-T therapy with small molecule drugs such as venetoclax (BCL-2 inhibitors) and immune checkpoint inhibitors also offers significant potential for enhancing clinical efficacy (106).

### Applying dual targets to avoid on-target/off-tumor effects

Because most target antigens are present on both AML cells and healthy tissues, CAR-T cell therapy often causes on-target/off-tumor effects inevitably, even threatening the patient’s life. In order to avoid these toxicities in CAR-T cell treatment of AML, researchers have proposed many strategies. For example, a universal CAR platform technology known as UniCAR divides conventional CARs into two distinct elements: a CAR for the non-specific manipulation of T cells and a targeting module for redirecting the activity of UniCAR T cells. This strategy reduces the risk of on-target side effects by allowing the precise activation and deactivation of CAR-T cells in a regulated method (107). Logic-gated CAR also shows a promising prospect, such as AND gates incorporating two individual receptors, OR gates based on dual or tandem CARs, and NOT gates with inhibitory signaling. In a study, the CAR-T cells that dual-target CD13 and TIM3 showed great specificity for eradicating both CD13^pos^ and TIM3^pos^ AML cells, with acceptable toxicity to the healthy cells with only expressing CD13 (108). A recent study repurposed cytosolic molecules into CARs by combining the LAT with SLP-76, and generated the AND-gate CAR-T cells which exhibited enhanced functionality and specificity (109). Furthermore, researchers developed a new platform named AbTCR-CSR, which combined an antibody-T-cell receptor (AbTCR) CAR with costimulatory signaling receptor (CSR), representing a similar AND gates strategy to avoid the toxicity of CAR-T therapy in AML (110). In the context of NOT-gated CAR-T cell therapy, these cells are engineered to express a secondary inhibitory CAR (iCAR) designed to recognize an antigen exclusively expressed in healthy cells, not present in tumor cells. This iCAR delivers an inhibitory signal that effectively counteracts the activation signal intended for the CAR-T cells, thus modulating their responses to prevent unintended interactions with healthy tissues. Researchers have tested the NOT-gated CD93 CAR-T in an *in vitro* model with a rewarding outcome and promising prospect (46).

### Utilizing genetic engineering for CAR-T cells or healthy tissue cells

Gene editing can effectively avoid the T-cell exhaustion and enhance the activity of CAR-T cells. This strategy includes deleting negative regulatory molecules (111) or expressing specific transgenic molecules (112) to enhance anti-tumor activity, such strategy has been proved in many tumor models including AML. In addition, the gene editing strategy is also promising to avoid on-target/off-tumor toxicity. A study that deleted CD33 from normal HSCs in order to generate a hematopoietic system resistant to CAR-T cells showed the specific and effective killing capability of AML cells (64). However, the production of clinically compliant gene-edited CAR-T cells faces challenges including safety risks from off-target modifications causing genomic instability or oncogenic mutations, production efficiency concerns regarding CAR-T cell numbers and activity, and ethical issues in meeting global regulatory requirements (113, 114).

### Attaching polyethylene glycol to the exterior of CAR-T cells

CRS and neurotoxicity are recognized as the predominant and distinctive adverse effects linked to CAR-T cell therapies. A recent study has demonstrated that *in vivo* attachment of PEG to the exterior of CAR-T cells significantly alleviates the incidence of CRS and neurotoxicity which are commonly induced by CAR-T cells. Importantly, this modification does not influence the capacity of CAR-7 to eliminate tumors, therefore preserving their therapeutic efficacy. This is because the CAR-T cell can block the interactions between CAR-T cells, tumor cells and monocytes, and decrease both monocyte overactivation and cytokine release *in vitro*. Furthermore, the gradual proliferation of CAR-T cells decreases PEG surface density and facilitates the re-establishment of interactions between CAR-T cells and AML cells, leading to robust anti-tumor responses, while reducing adverse effects (115).

### Optimizing the production procedure of CAR-T

Despite some potential advantages, using autologous T cells to produce the CAR-T cells has complicated procedures which cost plenty of time, therefore prolonging the waiting time for treatment. The allogeneic FLT3 CAR-T cells is an off-the-shelf CAR-T therapy, showing the convenience and feasibility of, shortening the waiting time before treatment and increasing the odds for patients to acquire these therapies. Furthermore, the off-switch in the lead CAR mentioned in this study provides the possibility to modulate CAR-T cell activity and thus restores the functions of the hematopoietic system after the elimination of AML cells (49). Another limitation is that the intensive induction/salvage regimens, both for initial treatment and for rescuing purposes in AML, can significantly reduce the activity and the number of autologous T cells collected during leukapheresis. Consequently, producing CAR-T cells directly from AML patients would lead to the disappointing availability of CAR-T cells. Employing engineered CIK cell, genetically modified, as an allogeneic resource within the dual CAR strategy is promising for avoiding the decrease of autologous T cells activity (34).

**Figure 2:**
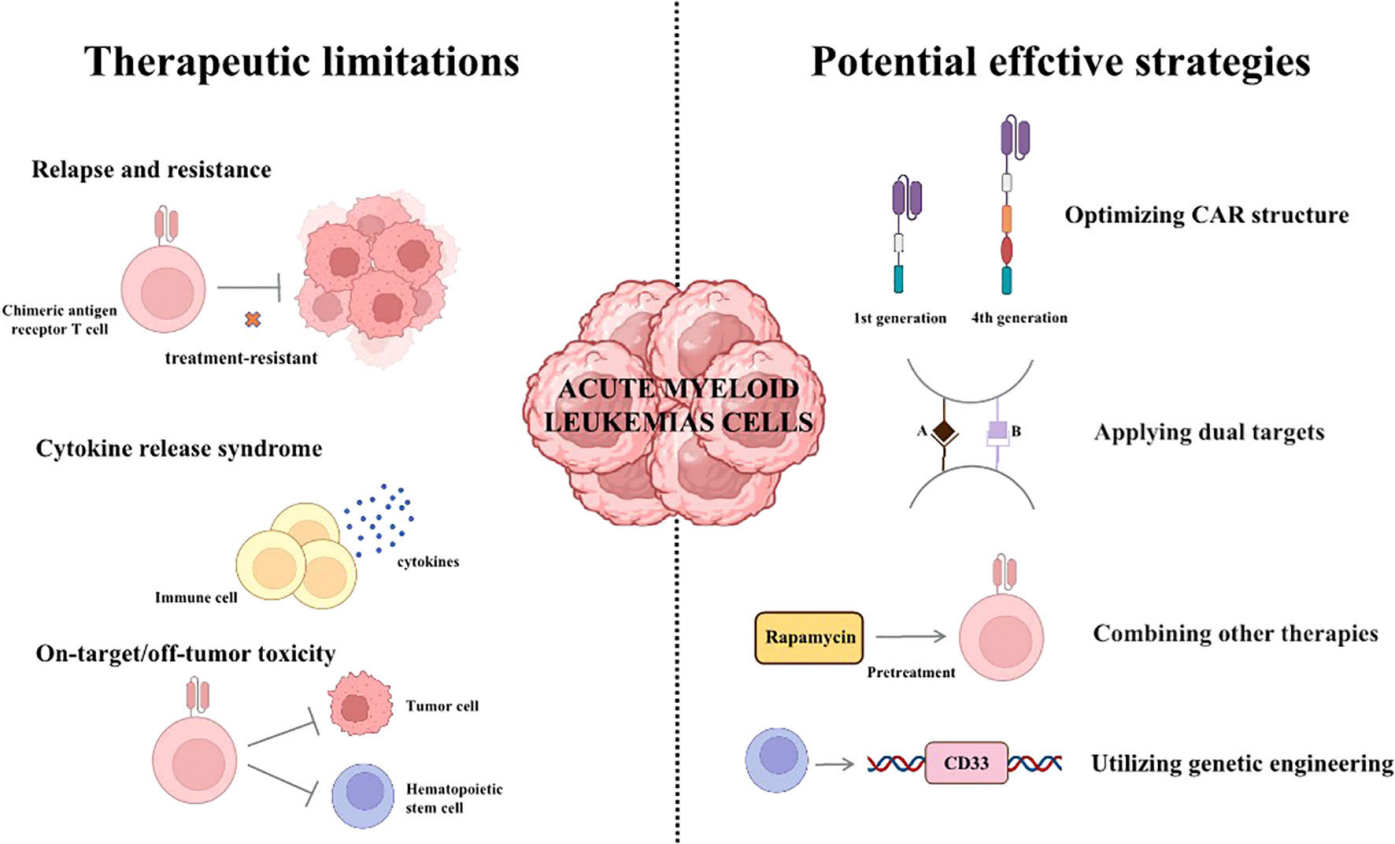
The therapeutic limitations and potential effective strategies of CAR-T therapy. (I) Relapse, resistance and adverse effects are significant challenges of CAR-T therapy currently. (II) Some strategies for overcoming the limitations of CAR-T therapy.

Despite the many limitations in CAR-T therapy for AML, researchers have demonstrated numerous strategies to deal with them (**Figure 2**). These existing strategies provide valuable insights and inspiration for the subsequent research. For example, developing more effective CAR structure may be significant potential for advancement. Combing other therapies, such as rapamycin for pre-treatment, is also rewarding for overcoming these challenges.

Translating these strategies into subsequent trials still faces numerous challenges. These challenges include the limitations of mouse models in accurately replicating the immunosuppressive and metabolic stress conditions of the human tumor microenvironment, the potential for relapse following treatment observed in clinical trials, the necessity for hematopoietic stem cell transplantation as a salvage therapy, the rapid disease progression characteristic of AML patients, and the intricate manufacturing process required for personalized CAR-T cell production. Collectively, these factors impose stringent requirements on the design and execution of subsequent clinical trials (116).

## Conclusions

Compared to recent systematic evaluations or meta-analyses (e.g., Shahzad et al., 2023) (14), we discuss in greater depth the many barriers and limitations faced by CAR-T cell therapies for the treatment of AML as well as strategies for avoiding adverse effects and improving the efficacy of CAR-T therapies. In this review, we outlined the progress achieved in the multiple categories of immunotherapeutic approaches for the management of AML, discussed the particular mechanisms of CAR-T therapy, and further summarized the recent advances of CAR-T immunotherapy in AML, as well as the current limitations, hopefully providing some novel insights for the future research direction.

Despite more and more treatment options in recent years, AML still poses a serious threat to human health with approximately 50% of patients ultimately dying from the disease progression and relapse. Immunological treatments, particularly CAR-T therapy, have shown magnificent efficacy in eliciting responses among patients with AML, indicating the likelihood of CAR-T to improve AML patient’s prognosis in future clinical practice. In the last several years, various CARs have been engineered and further evaluated rigorously in the clinical trials of AML patients to clarify their safety and efficacy. In addition, significant advancements have been achieved in overcoming the resistance of R/R AML and avoiding CAR-T cell-associated adverse toxicities. Although the CAR-T therapy for AML remains immature compared with other hematological malignancies such as ALL, we believe that CAR-T therapy holds great potential benefits for AML patients in the future.

At present, CAR-T cells still display numerous limitations to be overcome in order to improve the prognosis of AML patients. The foremost challenge among these issues is the safety of CAR-T therapy, which needs further input into the better design of CAR and avoiding the cross-interaction of both AML cells and health tissues, thus emphasizing the importance of optimization of CAR structures or combination of CAR with other therapies. Resistance and relapse of AML are also a sticky challenge, therefore clarifying the genetic and phenotypic heterogeneity of AML cells and utilizing gene editing technology to modify the CAR-T cells might be highly appreciated. The combination of multiple strategies may be even the futural dominant approach. All in all, in spite of the limitations, CAR-T therapy enriches the toolbox of AML treatment currently and is worthy of much great research in the future.
